# Development of a mouse model for *Klebsiella pneumoniae*-associated neonatal sepsis

**DOI:** 10.1128/spectrum.00697-25

**Published:** 2025-08-01

**Authors:** Jernelle C. Miller, Myeongjin Choi, Zhiyong Zhao, Ember M. Mushrush, Teklu B. Legesse, Alan S. Cross, Scott M. Baliban, Sharon M. Tennant

**Affiliations:** 1 Center for Vaccine Development and Global Health, University of Maryland School of Medicine 12264, Baltimore, Maryland, USA; 2 Department of Medicine, University of Maryland School of Medicine 12264, Baltimore, Maryland, USA; 3 Department of Pathology, University of Maryland Medical Center 21668, Baltimore, Maryland, USA; Houston Methodist, Houston, Texas, USA

**Keywords:** *Klebsiella pneumoniae*, neonatal sepsis, bacterial virulence, immune response, animal infection models, vaccine

## Abstract

*Klebsiella pneumoniae* is a major cause of neonatal sepsis in low- to middle-income countries. With the proportion of multidrug-resistant *K. pneumoniae* strains increasing globally and the lack of novel antibiotics in the pipeline, vaccination to prevent infections due to *K. pneumoniae* is an alternative strategy to antibiotics. Several *K. pneumoniae* vaccines are currently in development; however, there are no small animal models of *K. pneumoniae*-associated neonatal sepsis that can be used to evaluate vaccine efficacy. Therefore, we developed and characterized a neonatal murine model of *K. pneumoniae* infection. We found that neonatal C57BL/6 mice were highly susceptible to *K. pneumoniae* B5055, a serotype O1:K2 hypervirulent strain, via peroral inoculation, and the susceptibility of mice to *K. pneumoniae* infection was age and dose dependent. Mice aged 2–3 days consistently exhibited 88%–100% mortality when infected with 10^8^–10^9^ colony-forming units of *K. pneumoniae* B5055. This susceptibility progressively decreased during the neonatal period such that *K. pneumoniae* B5055 at this dose was avirulent in 10-day-old and older C57BL/6 mice following peroral administration. Two-day-old mice were bacteremic as early as 2 h post-infection, which was accompanied by systemic dissemination of *K. pneumoniae* beyond the gastrointestinal tract into the brain, liver, lungs, and spleen. However, only the liver and lungs displayed inflammatory infiltrate around the portal tract and neutrophilic exudate in the alveoli and air spaces, when compared to uninfected mice, which showed no pathological effects. Collectively, these results show that we have generated a novel and reproducible animal model for *K. pneumoniae* neonatal sepsis.

**IMPORTANCE:**

The development of appropriate vaccines for *K. pneumoniae* relies on suitable animal models for *in vivo* efficacy testing. However, to date, there are no small animal models of *K. pneumoniae*-associated neonatal sepsis. We have established a neonatal mouse model of lethal *K. pneumoniae* infection that is age dependent and mimics the heightened susceptibility to *Klebsiella* spp. observed in human neonates. This newly discovered mouse model represents a valuable tool to study the pathogenesis of invasive *K. pneumoniae* infections in the neonate and to develop novel vaccines aimed at minimizing morbidity and mortality associated with *K. pneumoniae* neonatal sepsis.

## INTRODUCTION

*Klebsiella pneumoniae* is an opportunistic healthcare-associated pathogen that is responsible for a substantial burden of bacterial pneumonia and invasive disease worldwide (1, 2). Recently, *K. pneumoniae* has been implicated as a common cause of bloodstream infections and sepsis in neonates (3, 4). Neonatal sepsis is defined as a systemic inflammatory response occurring in infants ≤28 days of life and is commonly caused by multiple bacterial pathogens (5–8). A systematic review found that *Klebsiella* spp., *Escherichia coli*, *Enterobacter* spp., and *Pseudomonas* spp. accounted for 38% of blood culture-positive neonatal sepsis cases in sub-Saharan Africa (9). Other studies, including the Child Health and Mortality Prevention Surveillance (CHAMPS), the Burden of Antibiotic Resistance in Neonates from Developing Societies, and a global neonatal sepsis observation cohort study, have implicated *K. pneumoniae* as one of the predominant causative pathogens associated with neonatal sepsis and death in low- to middle-income countries (LMICs) (10–13). High rates of antimicrobial-resistant *K. pneumoniae*, in addition to poor accessibility and affordability of appropriate antibiotics in LMICs, have been reported (10, 14, 15). Additionally, the World Health Organization considers multidrug-resistant *K. pneumoniae* to be a critical threat (16). Multiple groups are developing *K. pneumoniae* vaccines for various clinical indications, including prevention of neonatal sepsis (17–19). However, no vaccines that target *K. pneumoniae* have yet been licensed.

Sepsis in neonates is often divided into early- and late-onset forms. In general, early-onset neonatal sepsis is thought to be the result of maternal transmission of invasive pathogens during the perinatal period, whereas late-onset sepsis results from environmental exposure to pathogens post-natally (7, 20–23). Establishing early protection from *K. pneumoniae* might significantly reduce the morbidity and mortality associated with both early- and late-onset neonatal sepsis. Therefore, maternal immunization affords the optimal opportunity to prevent neonatal sepsis via the transfer of protective maternal antibodies to neonates or blockage of *K. pneumoniae* transmission from mothers to their children. Recent modeling data suggest that a maternal *K. pneumoniae* vaccine with ~70% efficacy could avert approximately 80,000 neonatal deaths and 400,000 neonatal sepsis cases yearly worldwide (10).

Adult rodents are the model of choice to investigate *K. pneumoniae*-induced respiratory, gastrointestinal (GI), and urinary tract infections (24). For *K. pneumoniae* respiratory infection rodent models, several methods of inoculation (e.g., intranasal inoculation and intratracheal inoculation with or without surgery) have been described that effectively promote lung disease and show evidence of increased bacterial load in the lung and systemic dissemination (24). *K. pneumoniae* GI infection models primarily utilize oral administration to mimic natural acquisition, but these models generally require pre-administration of antibiotics to clear the gut microbiota (24). In terms of eliciting lethal bacteremia in adult mice, we and others have used the intravenous or intraperitoneal (i.p.) routes of infection (25, 26). In an elegant study, Russo et al. used a bacteremia mouse model incorporating intraperitoneal or subcutaneous (s.c.) injection to examine the virulence of classical and hypervirulent *K. pneumoniae* isolates (27). Despite the utility of adult rodent models for understanding *K. pneumoniae* disease pathogenesis, they cannot effectively mimic *K. pneumoniae*-associated neonatal sepsis due to age-related differences of the host. This is a critical knowledge gap. A reliable neonatal sepsis model would advance our knowledge of *K. pneumoniae* infections and help to develop strategies that mitigate the risk of morbidity and mortality associated with neonatal sepsis.

Animal models are routinely used to evaluate vaccine efficacy. To protect neonates against infection, since there is often insufficient time to elicit an immune response, vaccines may need to be administered maternally to confer passive protection to their offspring. Rodent neonatal sepsis models for group B *Streptococcus* (GBS) and *E. coli* have been developed; maternally administered vaccines have been shown to confer protection to pups following lethal challenge with GBS (28, 29). Additionally, maternal antibodies elicited by a live attenuated *E. coli* K1 vaccine have conferred protection in neonates following lethal challenge with both K1 and non-K1 strains of *E. coli* (30, 31). Collectively, these studies highlight the promise of maternal immunization in eliciting protective immunity against lethal bacterial challenge using a relevant neonatal sepsis animal model.

In this work, we developed a *K. pneumoniae*-associated neonatal sepsis murine model using the well-studied B5055 (O1:K2) hypervirulent strain (32). We evaluated various strains of mice and bacterial isolates, routes of infection, and susceptibility to infection by age and assessed bacterial loads in tissues, as well as histopathological effects early and late in infection. This novel model system provides a useful tool to examine host-microbial interactions during *K. pneumoniae* bloodstream infection in newborns as well as to develop countermeasures against *K. pneumoniae* neonatal sepsis.

## RESULTS

### Comparison of the sensitivity of different mouse strains to *K. pneumoniae* B5055 Strep^R^

**Fig 1:**
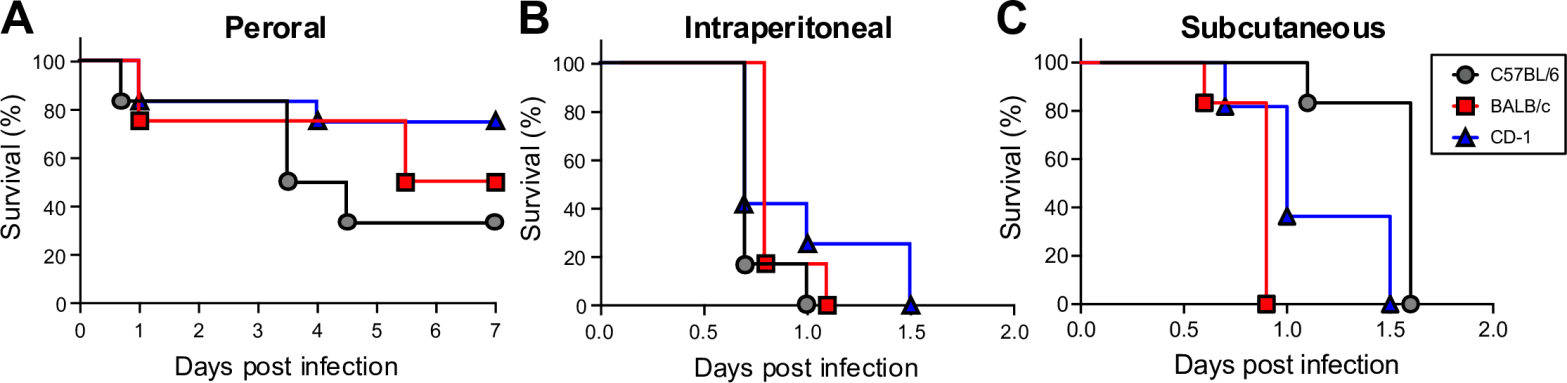
Survival comparison of neonatal mouse strains infected with *K. pneumoniae* B5055. Whole litters of 2-day-old C57BL/6 mice (circles, gray lines), BALB/c mice (squares, red lines), or CD-1 mice (triangles, blue lines) were infected with *K. pneumoniae* B5055 Strep^R^ either (**A**) perorally, with 4.2–6.8 × 10^8^ CFU (*n* = 4–12 per group); (**B**) intraperitoneally, with 0.8–1.3 × 10^6^ CFU; or (**C**) subcutaneously, with 0.8–1.3 × 10^6^ CFU (*n* = 4–12 per group). Mice were monitored daily for survival. The p.o. infection experiments were performed at least two times, and the Kaplan-Meier survival curves shown here are from one representative experiment. The Kaplan-Meier survival curves shown for i.p. and s.c. infection experiments are a single experiment for each administration route.

To compare the susceptibility of different mouse strains to *K. pneumoniae* infection and the effect of administration route on susceptibility, 2-day-old BALB/c, C57BL/6, and CD-1 mice were infected either perorally, intraperitoneally, or subcutaneously with a streptomycin-resistant strain, *K. pneumoniae* B5055 Strep^R^. Use of streptomycin resistance allowed us to selectively enumerate *K. pneumoniae in vitro*. Two-day-old mice were selected to model early-onset sepsis. All animals were monitored daily for clinical signs of sepsis (see Table 2), and all remaining surviving animals were euthanized 7 days post-infection (d.p.i.). For peroral (p.o.) administration, mice were infected with 4.2–6.8 × 10^8^ colony-forming units (CFU). C57BL/6 mice exhibited 33% (2 out of 6) survival, while BALB/c and CD-1 mice exhibited 50% (2 out of 4) and 75% (9 out of 12) survival, respectively (Fig. 1A). Perorally infected mice displayed a mean time to death (MTD) of 5.3 ± 2.4 d.p.i. (± standard deviation). All animals were susceptible to *K. pneumoniae* B5055 Strep^R^ following i.p. and s.c. administration of 0.8–1.3 × 10^6^ CFU, succumbing to infection within a MTD of 1.0 ± 0.32 d.p.i. (Fig. 1B and C). Representative data are shown in Fig. 1. To examine whether there were any differences in MTD for the peroral route between mouse strains, we combined data from two experiments. We observed an MTD of 3.47 days for C57BL/6 mice (9 mice died out of 11 mice tested), 4.1 days for BALB/c mice (6 mice died out of 10 mice tested), and 5.04 days (11 mice died out of 24 mice tested) for CD-1 mice. There was a statistically significant difference (*P* ≤ 0.05) in the MTD between C57BL/6 and CD-1 mice. Collectively, these data suggest that for the i.p. and s.c. administration routes, all three mouse strains responded similarly to *K. pneumoniae* infection, while neonatal C57BL/6 mice were more susceptible to p.o. infection.

**Fig 2:**
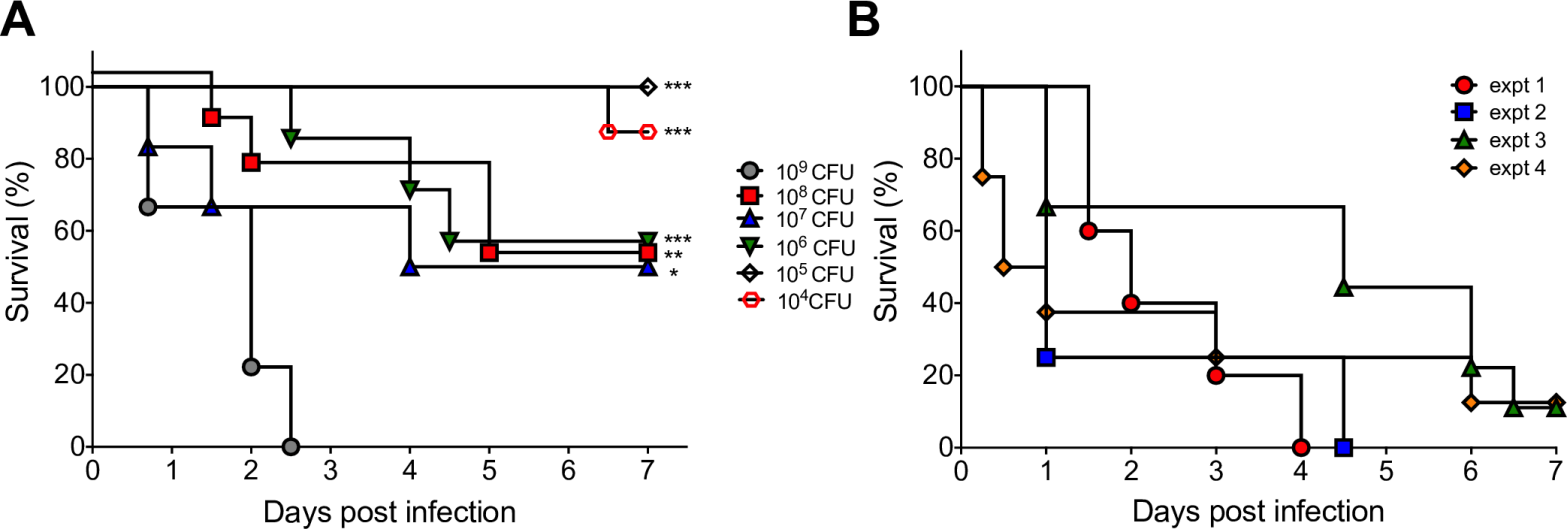
The repeatability and stability of the *K. pneumoniae*-associated neonatal mouse infection model. (**A**) Whole litters of 2- or 3-day-old C57BL/6 mice (*n* = 5–9 per litter) were infected perorally with the indicated inocula of *K. pneumoniae* B5055 Strep^R^ and monitored daily for survival to determine the 50% lethal dose. Statistical survival differences were determined by log-rank analysis relative to 2.2 × 10^9^ CFU. *, *P* ≤ 0.01; **, *P* ≤ 0.001; ***, *P* ≤ 0.0001. (**B**) Whole litters of 2-day-old C57BL/6 mice (*n* = 5–9 per litter) were infected perorally in four independent experiments (expt) with 1.5 × 10^9^ CFU (expt 1), 6.0 × 10^8^ CFU (expt 2), 6.0 × 10^9^ CFU (expt 3), and 1.4 × 10^9^ CFU (expt 4) of *K. pneumoniae* B5055 Strep^R^ and monitored daily for survival to evaluate the reproducibility of the neonatal mouse model.

To determine the peroral 50% lethal dose (LD_50_), 2- or 3-day-old CD-1 mice (*n* = 6–15 per group) and C57BL/6 mice (*n* = 5–9 per group) (BALB/c mice not tested) were infected perorally with serially diluted *K. pneumoniae* B5055 Strep^R^. CD-1 mice exhibited 77%–89% survival at infection doses ranging from 6.8 × 10^5^ CFU to 6.8 × 10^8^ CFU, indicating *K. pneumoniae* B5055 Strep^R^ was marginally virulent following peroral infection (LD_50_ >6.8 x 10^8^ CFU) (Fig. S1). In contrast, all neonatal C57BL/6 mice infected with a dose of 2.2 × 10^9^ CFU per mouse died within 2.5 d.p.i. (zero out of nine survived), while 50% (four out of eight), 50% (three out of six), 57.1% (four out of seven), 100% (five out of five), and 87.5% (seven out of eight) survival was observed for mice infected with 2.2 × 10^8^ CFU, 2.2 × 10^7^ CFU, 2.2 × 10^6^ CFU, 2.2 × 10^5^ CFU, and 2.2 × 10^4^ CFU, respectively (Fig. 2A). We calculated the p.o. LD_50_ of *K. pneumoniae* B5055 Strep^R^ to be 8.5 × 10^6^ CFU in 2- to 3-day-old C57BL/6 mice. We subsequently determined the LD_50_ for the i.p. and s.c. routes in CD-1 and C57BL/6 mice (BALB/c not tested) to be <100 CFU (Fig. S2 and S3). Since the minimum lethal dose for *K. pneumoniae* B5055 Strep^R^ following i.p. and s.c. administration was low (~10 CFU), we selected the p.o. infection route and C57BL/6 mice for subsequent experiments to enhance the reproducibility of the model.

To ensure that a consistent attack rate was achievable with ~10^9^ CFU of *K. pneumoniae* B5055 Strep^R^, which produced 100% lethality in neonatal C57BL/6 mice, we evaluated survival in four independent experiments. Two-day-old C57BL/6 mice (*n* = 5–9 mice per experiment) were infected perorally with either 1.5 × 10^9^ CFU (experiment 1), 6.0 × 10^8^ CFU (experiment 2), 6.0 × 10^9^ CFU (experiment 3), or 1.4 × 10^9^ CFU (experiment 4). We observed 0% (zero out of five) survival, 0% (zero out of eight) survival, 11% (one out of nine) survival, and 12.5% (one out of eight) survival in experiments 1, 2, 3, and 4, respectively. These data demonstrated that an inoculum of at least 6.0 × 10^8^ CFU could reliably produce 88%–100% mortality in neonatal C57BL/6 mice (median survival of 5.55% ± 5.92%) (Fig. 2B).

To determine if other strains of *K. pneumoniae* were capable of establishing infection in neonatal mice, we assessed several clinical *K. pneumoniae* isolates following p.o. administration. Two-day-old C57BL/6 mice were infected perorally with 1.0 × 10^8^ to 2.2 × 10^9^ CFU of TPEVGH-KPN-12 (O2:K2), 700603-MP (O3:K undetermined), 390 (O3:K11), 15AP507624 (O5:K14), 12-02000 (O5:K53), or 4425/51 (O5:K57). We observed 0% survival in neonatal mice infected with TPEVGH-KPN-12 (zero out of three), 700603-MP (zero out of three), and 15AP507624 (zero out of six), while 12-02000, 4425/51, and 390 exhibited 50% (three out of six), 80% (four out of five), and 100% (six out of six) survival, respectively (Fig. S4). These results highlight the intrinsic variability in virulence among *K. pneumoniae* isolates to cause mortality in a neonatal sepsis model following p.o. administration.

### Examination of age-dependent peroral infection with *K. pneumoniae* B5055 Strep^R^

**Fig 3:**
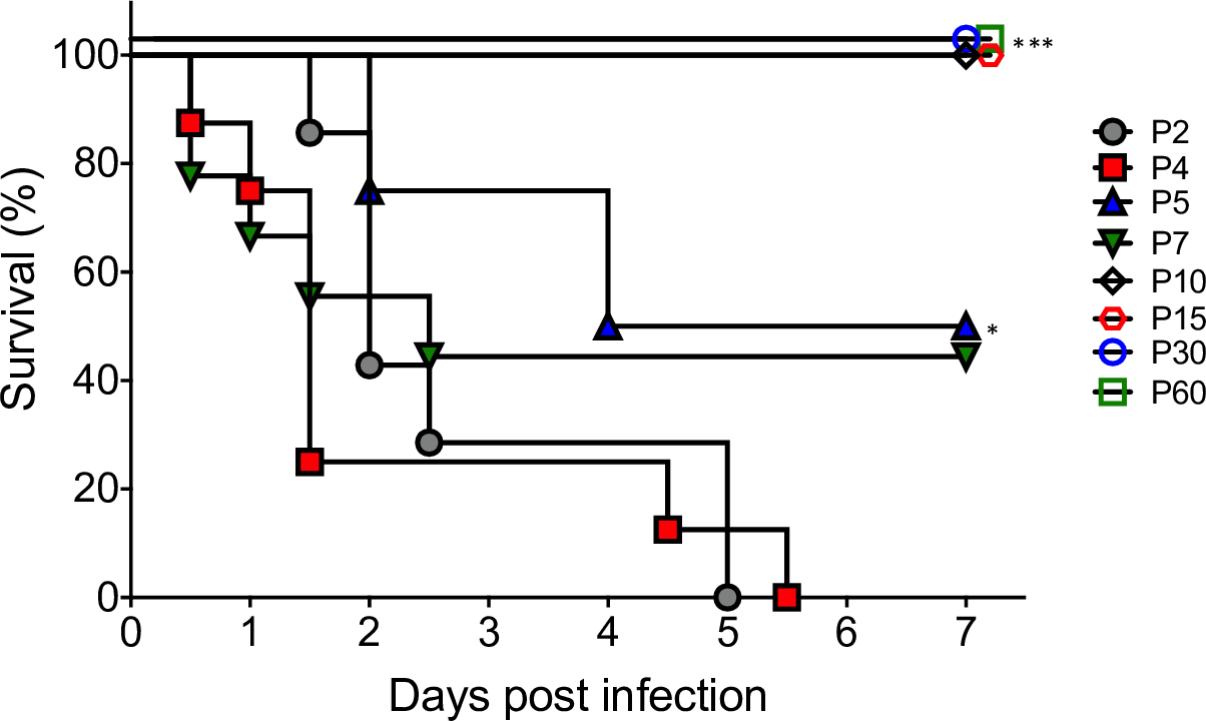
*K. pneumoniae* B5055 Strep^R^ elicits an age-dependent effect following peroral infection. C57BL/6 mice at 2, 4, 5, 7, 10, 15, 30, and 60 days of age (*n* = 5–9 per age group) were infected perorally with *K. pneumoniae* B5055 Strep^R^ (1.4–2.0 × 10^8^ CFU/mouse) and monitored daily for survival. Data are presented as Kaplan-Meier survival curves. Results shown are from one representative experiment performed at least two times. Statistical survival differences were determined using a log-rank analysis relative to P2. *, *P* ≤ 0.05; ***, *P* ≤ 0.0001.

To assess age susceptibility to *K. pneumoniae* infection, C57BL/6 mice at 2, 4, 5, 7, 10, 15, 30, and 60 days of age were infected perorally with 1.4–2.0 × 10^8^ CFU *K*. *pneumoniae* B5055 Strep^R^. Two- and 4-day-old mice exhibited 0% survival within 5.5 d.p.i. (zero out of seven and zero out of eight, respectively) (Fig. 3). Five- and 7-day-old mice exhibited 50% (four out of eight) and 44% (four out of nine) survival, respectively. In contrast, 10-, 15-, 30-, and 60-day-old mice were not susceptible to infection with *K. pneumoniae*, with 100% survival at 1.4 × 10^8^ CFU. These results suggest that susceptibility to *K. pneumoniae* declines with age and that animals 4 days of age or younger are the most sensitive to *K. pneumoniae* infection following p.o. administration.

### Evaluation of bacterial burden following peroral infection with *K. pneumoniae* B5055 Strep^R^ in neonatal mice

**Fig 4:**
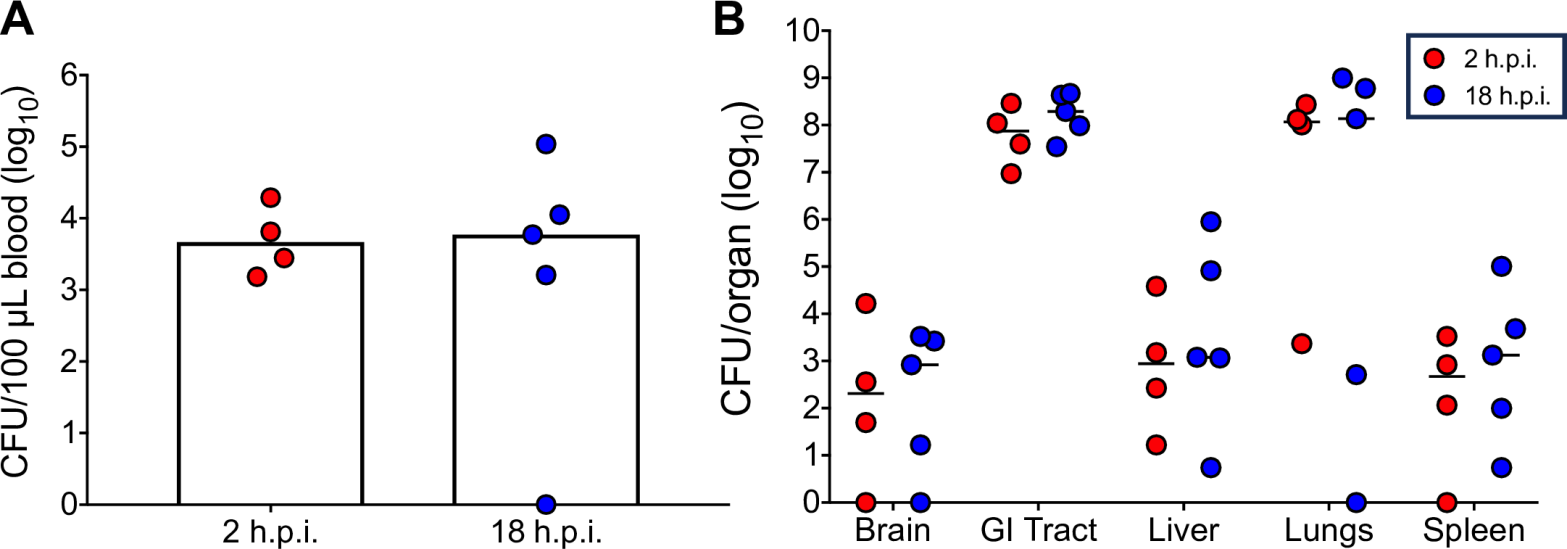
Tissue bacterial burden in *K. pneumoniae-*infected neonatal mice. Two-day-old C57BL/6 mice (*n* = 4–5 per timepoint) were infected perorally with 1.3–3.3 × 10^7^ CFU of *K. pneumoniae* B5055 Strep^R^. Bacterial loads in the (**A**) blood and (**B**) tissues (brain, intestine, liver, lungs, and spleen) were determined at two or 18 h post-infection (h.p.i.). Each point represents an individual mouse. Results shown are one representative experiment performed at least two times. Median is represented by the bar.

To understand the temporal dynamics of *K. pneumoniae* infection in neonatal mice, we harvested blood and various tissues (brain, GI tract, liver, lungs, and spleen) and counted viable bacteria at 2 and 18 h post-infection (h.p.i.), which both are prior to the expected onset of mortality at 24 h.p.i. (Fig. 2B). Two-day-old C57BL/6 mice were perorally infected with 1.3–3.3 × 10^7^ CFU of *K. pneumoniae* B5055 Strep^R^. *K. pneumoniae* was detected in the blood and tissues as early as 2 h.p.i., with no significant difference of *K. pneumoniae* observed in the blood, brain, GI tract, liver, lungs, and spleen between 2 and 18 h.p.i. (Fig. 4A and B). As expected, the GI tract contained high loads of *K. pneumoniae*. Interestingly, the lungs also contained high loads of *K. pneumoniae*, ~4 log_10_ CFU higher, compared to the bacterial load in the brain, liver, and spleen (Fig. 4B). To assess whether the gavage technique was contributing to the pattern of *K. pneumoniae* dissemination after local infection of the GI tract, neonatal mice were inoculated perorally with either a 20- or 24-gauge feeding needle with 1.5 × 10^7^ CFU. The 20-gauge feeding needle allows for delivery into the oral cavity, whereas the 24-gauge needle allows for a more precise delivery to the back of the throat at the top of the esophagus. Following inoculation, neonatal mice were euthanized at 2 h.p.i., and bacterial burden was evaluated. We observed no significant differences between colonization, across tissue type, between 20- and 24-gauge infected neonates (Fig. S5). We found that it was easier to infect neonates using the 24-gauge gavage needle and used this method for subsequent experiments.

### Assessment of pathology following peroral infection with *K. pneumoniae* B5055 Strep^R^ in neonatal mice

**Fig 5:**
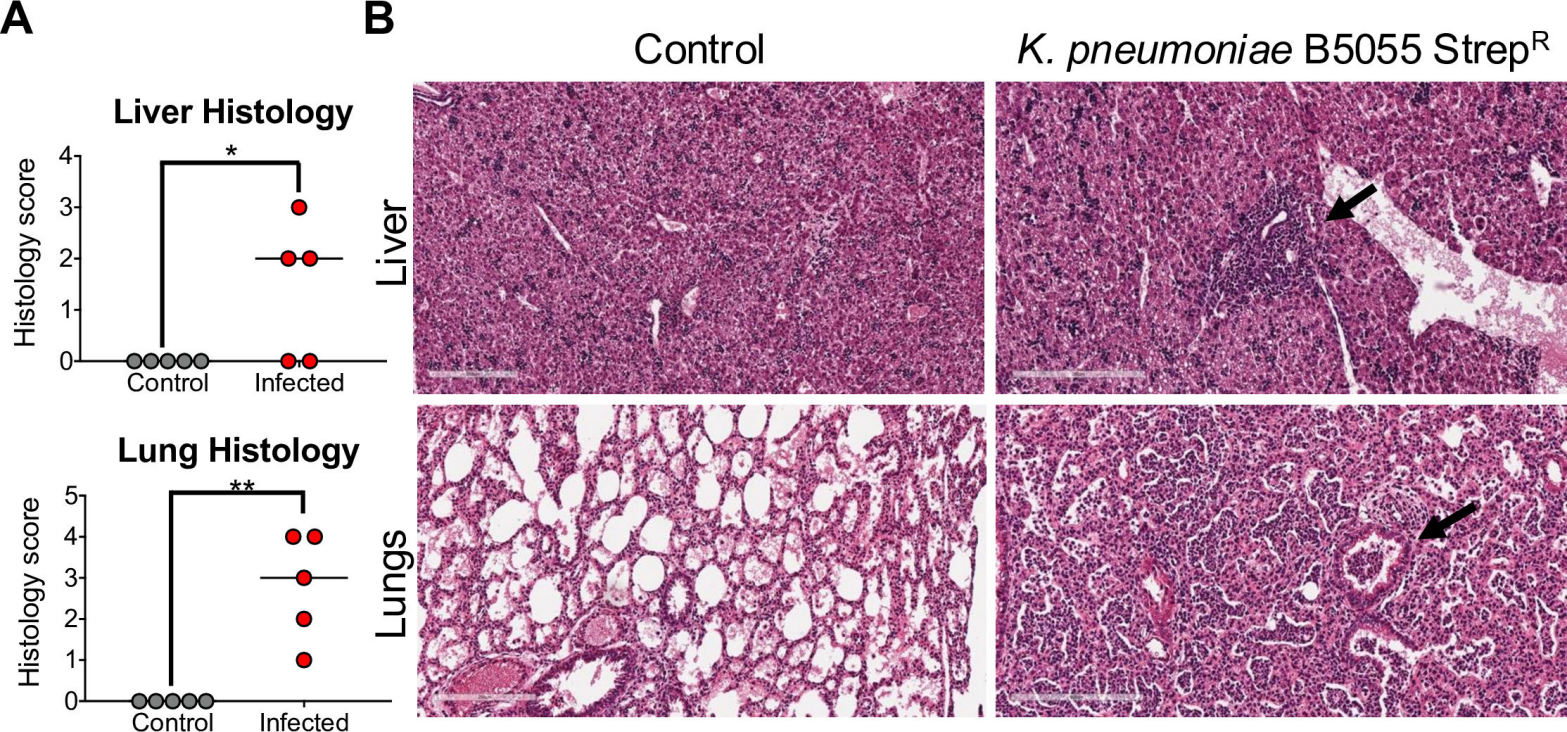
*K. pneumoniae* B5055 Strep^R^ induces systemic pathology in neonatal mice. (**A**) Liver and lung pathologies were scored in *K. pneumoniae* B5055 Strep^R^-infected and control 2-day-old C57BL/6 mice (*n* = 5 per group) at 18 h.p.i. Degree of pathology was based on Table 2, with a maximum score of 8. Data points represent an individual mouse from a single experiment (*, *P* ≤ 0.05; **, *P* ≤ 0.001, by Student’s *t*-test). (**B**) Representative images (magnification ×200, scale bar 200 µm) of hematoxylin and eosin-stained liver and lung sections from neonatal mice at 18 h.p.i. following peroral infection with *K. pneumoniae* B5055 Strep^R^ (6.5 × 10^7^ CFU/mouse). Arrows indicate the location of liver inflammatory infiltrate around the portal tract and lung neutrophilic exudate in the alveoli and air spaces. Images for *K. pneumoniae* infection are from mice which exhibited pathology, scoring 3 for the liver and 4 for the lungs.

Having recovered *K. pneumoniae* from the brain, GI tract, liver, lungs, and spleen of infected neonates, we examined whether this dissemination was associated with pathology in these organs. Two-day-old C57BL/6 mice were perorally infected with 6.5 × 10^7^ CFU *K*. *pneumoniae* B5055 Strep^R^, and tissues were harvested at 18 h.p.i. We observed no histopathological effects in the brain, GI tract, and spleen (Fig. S6), despite bacterial burden in these tissues, while significant inflammation was observed in the liver and lungs of infected neonates at 18 h.p.i. when compared to control Evans blue dye-inoculated mice (Fig. 5A). *K. pneumoniae*-infected neonates displayed inflammatory infiltrates around the portal tract in the liver (Fig. 5B, arrows) and neutrophilic exudate in the alveoli and air spaces, when compared to control Evans blue dye-inoculated mice (Fig. 5B).

## DISCUSSION

In this study, we describe a model of *K. pneumoniae*-associated neonatal sepsis using 2- to 3-day-old C57BL/6 mice, which showed reliable and consistent susceptibility to *K. pneumoniae* following peroral administration. In addition, C57BL/6 mice displayed an age-dependent response to infection and showed increased inflammatory infiltration into the portal tract of the liver and alveolar spaces of the lungs. This is consistent with observed clinical characteristics of septicemic infants, as studies have demonstrated sepsis-associated liver injury is associated with a higher risk of mortality, and lung injury can be induced by sepsis yielding greater complications, including mortality (33–36).

We found that neonatal mice were highly susceptible to *K. pneumoniae* infection, in contrast to previous reports using adult mice. Russo et al. infected 4- to 6-week-old CD-1, BALB/c, or C57BL/6 mice by the intraperitoneal or subcutaneous routes (27). Classical *K. pneumoniae* was only lethal at high doses (~10^8^ CFU), whereas hypervirulent strains produced an LD_50_ of 10^3^–10^5^ CFU, with mice taking 2–10 days to die. In contrast to these results observed in adult mice, we found that neonatal C57BL/6, BALB/c, and CD-1 mice were highly susceptible to intraperitoneal or subcutaneous injection with a hypervirulent *K. pneumoniae* isolate, with mice dying within 1.5 days post-infection. Additionally, the LD_50_ in neonatal CD-1 and C57BL/6 mice was <100 CFU. Peroral colonization models have also been described for adult mice, but these generally require pre-administration of antibiotics to clear the gut microbiota and circumvent colonization resistance (24). Our results corroborated these previous studies; we observed that C57BL/6 mice aged 10 days or older were resistant to infection with a hypervirulent strain of *K. pneumoniae*. In contrast, neonatal C57BL/6, BALB/c, and CD-1 mice were naturally susceptible to peroral infection with hypervirulent *K. pneumoniae* and did not require antibiotic pre-treatment. The peroral LD_50_ of *K. pneumoniae* B5055 Strep^R^ in C57BL/6 neonates was determined to be 8.5 × 10^6^ CFU. Therefore, our neonatal rodent model, unlike current adult models, is capable of establishing robust infection following peroral administration of *K. pneumoniae*, which we believe is the natural route of acquisition (24). Our data suggest a heightened susceptibility of newborn mice to *K. pneumoniae* infection that declines rapidly with age. This observation highlights how the normal aging process impacts susceptibility to *K. pneumoniae*, as has also been observed when comparing pulmonary infection of adult and aged immunosenescent mice (37).

We found that *K. pneumoniae* B5055 Strep^R^ infection consistently produced mortality in 2- to 3-day-old C57BL/6 mice when administered perorally, with *K. pneumoniae* found in tissues as early as 2 h.p.i. (Fig. 4A). *K. pneumoniae* is a leading cause of hospital-acquired infections with colonization and infection driven by environmental contamination, antibiotic selection, and person-to-person transmission (2, 38–40). Neonatal acquisition of *K. pneumoniae* is believed to occur during the perinatal period, via mother-to-fetus transmission during pregnancy, passage through the birth canal during childbirth, or breastfeeding (41–43). Gut colonization with *K. pneumoniae* can be a crucial precursor to bloodstream infection and the development of neonatal sepsis, highlighting the clinical relevance of using peroral administration to study early stages of infection (44, 45). Furthermore, analysis of the CHAMPS data, which identified *K. pneumoniae* as a contributory pathogen in child deaths, determined that 21% of child deaths had *K. pneumoniae* in the causal chain of death (13, 46). The most common clinical syndrome among *K. pneumoniae*-related chain deaths in children from this study was sepsis (44%), with *K. pneumoniae* colonizing the blood, heart, lung, liver, and cerebrospinal fluid (46). Our mouse model therefore mimics the early acquisition of *K. pneumoniae*, colonization, and dissemination seen clinically in human neonates.

Human neonates and the elderly are at increased risk of *K. pneumoniae* infection due to their immature or aging immune responses, respectively, suggesting an age-dependent response to infection (2). Our data corroborate these clinical findings, as adolescent and adult mice were not susceptible to peroral infection with *K. pneumoniae* B5055 Strep^R^, in contrast to neonatal mice. This is consistent with other studies showing that adolescent and adult rodents were resistant to *E. coli* and Coxsackievirus infection, whereas neonatal rats or mice were highly susceptible to these pathogens following peroral and intracerebral administration, respectively (30, 47).

We used this optimized neonatal sepsis model to assess bacterial burden in neonatal mice following lethal infection. *K. pneumoniae* B5055 Strep^R^ colonized the GI tract and lungs and disseminated widely to secondary lymphoid tissues, brain, and liver as early as 2 h.p.i. In addition, severe pathology was observed in the liver and lungs of infected neonatal mice at 18 h.p.i., compared to uninfected neonatal mice. This pathology is consistent with the hyperinflammatory state that generally develops during sepsis in humans, where neutrophils are activated to produce toxic mediators, damaging the endothelium and alveolar epithelium (48–50). Recent work has also implicated hypervirulent *K. pneumoniae* as a cause of liver damage and abscesses in patients, particularly in Southeast Asia (51). These findings suggest that neonatal C57BL/6 mice infected with *K. pneumoniae* exhibit important clinical characteristics of disease.

This study has limitations. We optimized the neonatal sepsis model with a reference strain of *K. pneumoniae* belonging to serotype O1:K2 (B5055). Though commonly used in the literature to study *Klebsiella* pathogenesis, this strain may not be representative of neonatal bloodstream isolates. *K. pneumoniae* can be classified into two types, classical and hypervirulent, which primarily cause nosocomial or community-acquired infections, respectively, and can be distinguished via genotypic markers (2, 52). B5055 is a hypervirulent strain, and classical *K. pneumoniae* may behave differently in neonatal mice. Additionally, there is significant heterogeneity among *K. pneumoniae* isolates in terms of genome composition and virulence factor expression, which could theoretically have an impact on virulence in neonatal mice (53). However, we showed that other clinical *K. pneumoniae* isolates are capable of causing mortality in our model. Future work will be needed to determine if there is a correlation between virulence in neonatal mice and certain characteristics such as genetic loci associated with hypervirulence or other factors. Additionally, we did not evaluate the infection of neonates via the respiratory route, which is another important route of acquisition of *K. pneumoniae*.

In conclusion, we generated a new animal model for *K. pneumoniae* neonatal sepsis, which is reproducible and provides a strategy for studying microbial pathogenesis and host-microbe interactions. Future studies will expand this model to characterize *K. pneumoniae* isolates collected from neonates with sepsis and will assess protection of neonates against *K. pneumoniae* infection following maternal immunization with novel vaccines.

## MATERIALS AND METHODS

### Animals

To generate neonatal and juvenile mice, 8- to 10-week-old pregnant CD-1, BALB/c, and C57BL/6 mice were purchased at gestational day 14 from Charles River Laboratories (Wilmington, MA). Pups were delivered naturally at term gestation and remained with their dams until they reached the desired age, except for brief interruptions due to experimental procedures. Pups of both sexes were used for all experimental procedures. For experiments involving adolescent and adult mice, 3- to 4-week-old and 7- to 8-week-old female C57BL/6 mice were purchased from Charles River Laboratories and acclimated on-site for 7 days prior to bacterial challenge. BSL-2 containment was employed for all experiments involving live bacteria.

### Bacterial strains and medium

Bacterial strains used in this study are shown in Table 1. Streptomycin-resistant *K. pneumoniae* B5055 Strep^R^ was generated by passaging *K. pneumoniae* B5055 (a hypervirulent O1:K2 strain [32] originally obtained from Frits and Ida Ørskov, Statens Serum Institut, Denmark) on medium containing increasing concentrations of streptomycin and selecting resistant colonies. A mouse-passaged version of *K. pneumoniae* 700603 was generated by administering strain 700603 intraperitoneally to a CD-1 mouse and isolating bacteria 24 h later from the liver. The recovered strain (700603-MP) was verified to be *K. pneumoniae* through analytical profile index testing (bioMérieux, Craponne, France). All bacterial strains were maintained in animal-product-free Hy-Soy (HS) bacteriological media (10 g/L Soytone [Teknova, CA], 5 g/L Hy-yest [Kerry Bio-Science, Beloit, WI], and 5 g/L sodium chloride [American Bio, Natick, MA]) at 37°C. When needed, bacteriological agar (MilliporeSigma, Burlington, MA) was added at 15 g/L. For *K. pneumoniae* B5055 Strep^R^ bacterial recovery, the medium was supplemented with 50 µg/mL of streptomycin sulfate (Research Products International, Mt. Prospect, IL).

**TABLE 1:** *K. pneumoniae* strains used in this study

Strain	Characteristics (serotype)	Reference
B5055	Reference strain (O1:K2)	(32)
B5055 Strep^R^	Spontaneous streptomycin-resistant B5055 (O1:K2)	This study
TPEVGH-KPN-12	Clinical isolate, Taiwan (O2:K2)	(3)
700603-MP	Mouse-passaged version of American Type Culture Collection 700603 (O3:K undetermined)	This study
390	Clinical isolate, Germany (O3:K11)	(54)
15AP507624	Clinical isolate, Sweden (O5:K14)	(3)
12-02000	Clinical isolate, USA (O5:K53)	(3)
4425/51	Clinical isolate, Germany (O5:K57)	(54)

### Mouse infections

*K. pneumoniae* strains were recovered from cryopreservation on HS agar prior to inoculation in HS broth for overnight growth at 37°C, 220 revolutions per minute with aeration for 17.5 h. Bacteria were then washed twice by centrifugation at 3,000 × *g* for 10 min at 4°C using a Sorvall Legend XTR centrifuge, XT-1000 rotor (Thermo Fisher Scientific, Waltham, MA) followed by resuspension in sterile phosphate-buffered saline (PBS). Bacteria were diluted with sterile PBS to the desired CFU based on optical density at 600 nm. All bacterial inocula were confirmed by serial dilution and enumeration on HS agar.

To compare the sensitivity of the mouse strains and routes of administration, 2-day-old BALB/c, C57BL/6, and CD-1 mice were infected perorally, intraperitoneally, or subcutaneously with *K. pneumoniae* B5055 Strep^R^ suspended in PBS containing 0.5% weight/volume (wt/vol) Evans Blue Dye (MilliporeSigma) at the indicated doses. For p.o. infection, the inoculum was administered to mice using a fixed volume Eppendorf Research plus pipette (MilliporeSigma) attached to a disposable, flexible, polytetrafluoroethylene (PTFE) 20-gauge × 1.5″ feeding needle with a 2 mm ball on the tip (Braintree Scientific, Braintree, MA) in a 10 µL volume (Fig. S7A). For i.p. and s.c. infection, the inoculum was administered to mice in a 50 µL volume either in the lower right quadrant of the abdomen or the dorsal surface, respectively, using a 30-gauge × 5/16″ 1 mL insulin syringe (Covidien, Mansfield, MA). Immediately following infection, pups were monitored for signs of stress and marked with a pen for identification and returned to their cage. Mice were then monitored daily for symptoms associated with sepsis for 7 d.p.i. *K. pneumoniae*-associated sepsis symptoms were scored as per Table 2, and pups with a score ≥4 were considered to have met alternative endpoints and were euthanized and recorded as a death.

**TABLE 2:** Five-feature scoring system for clinical features of *K. pneumoniae*-associated neonatal sepsis

Parameters	Scoring for the listed parameters		
	Healthy (0)	Intermediate (0.5)	Unhealthy (1)
Color of the skin	Black color	–^*b*^	Gray/pale color
Righting reflex	Immediate reversing upon back placement	Difficulty reversing (>3 s) but eventually achieved	Cannot achieve reversing, remaining on backside
Stomach/milk line^*a*^	Visible	–	Not visible
Behavior	Active movement, huddling with siblings, feeding	Lethargic moments, slow to feed	Trembling, not within the nest, does not feed
Weight	Gain of 0.5–1.0 g/day	No weight gain	Weight loss

^*a*^ Milk spot not visible after 5 days post-infection. Neonates with scores of ≥4 were euthanized.

^*b*^ Dashes signify that the intermediate score was not applicable.

To assess the virulence of *K. pneumoniae* clinical isolates, 2-day-old C57BL/6 mice were infected perorally with either TPEVGH-KPN-12 (O2:K2), 700603-MP (O3:K undetermined), 390 (O3:K11), 15AP507624 (O5:K14), 12-02000 (O5:K53), or 4425/51 (O5:K57) suspended in PBS containing 0.5% wt/vol Evans Blue Dye (MilliporeSigma) at the indicated doses. The inoculum was administered to mice using a fixed-volume Eppendorf Research plus pipette (MilliporeSigma) attached to a disposable, flexible, PTFE 20-gauge × 1.5″ feeding needle with a 2 mm ball on the tip (Braintree Scientific) in a 10 µL volume. Neonatal mice were observed daily for signs of sepsis as per Table 2 and mortality until 7 d.p.i.

For age dependency experiments, C57BL/6 mice at 2, 3, 4, 5, 7, 10, 15, 30, and 60 days of age were infected perorally with *K. pneumoniae* B5055 Strep^R^ (1.4–2.0 × 10^8^ CFU per mouse suspended in PBS containing 0.5% [wt/vol] Evans Blue Dye). For mice aged 15 days or younger, the inoculum was administered via a sterile, flexible PTFE 20-gauge × 1.5″ feeding needle with 2 mm ball in a 10 µL volume. For 30- and 60-day-old mice, the inoculum was administered via a sterile, straight, stainless steel 20-gauge × 1.5″ feeding needle with 2 mm ball (Cadence Science, Cranston, RI) in a 100 µL volume. Neonatal and infant mice (2–10 days old) were observed daily for signs of sepsis as per Table 2 and mortality until 7 d.p.i. Adolescent and adult mice (15–60 days old) were observed daily for alternative endpoints (weight loss, decrease in activity, and labored breathing) until 7 d.p.i. All experiments were performed at least twice, with representative data from single experiments presented.

### Quantitative bacteriology

Two-day-old C57BL/6 mice were infected perorally with *K. pneumoniae* B5055 Strep^R^ suspended in PBS containing 0.5% (wt/vol) Evans Blue Dye (1.3–3.3 × 10^7^ CFU per mouse) or PBS containing 0.5% (vol/vol) Evans Blue dye alone (control). Mice were manually restrained, and the inoculum was administered via a sterile, flexible, PTFE 20-gauge-1.5″ 2 mm ball feeding needle or with a sterile, straight, stainless steel 24-gauge-1″ 1.25 mm ball feeding needle (Cadence Science) in a 10 µL volume (Fig. S7B). At 2 or 18 h post-infection (h.p.i.), the brain, GI tract, liver, lungs, and spleen were excised aseptically, weighed, and homogenized in PBS using an Omni International TH-01 tissue homogenizer (Swedesboro, NJ). Blood was extracted following euthanasia into EDTA/KE microtubes (Sarstedt, Numbrecht, Germany). Tissue homogenates and blood were serially diluted in PBS and spread plated on HS containing streptomycin to determine the *K. pneumoniae* B5055 Strep^R^ load in tissues. Following incubation at 37°C overnight, colonies were counted and data were presented as CFU per gram of organ or CFU per 100 µL blood.

### Tissue histopathology

Two-day-old C57BL/6 mice were infected with *K. pneumoniae* B5055 Strep^R^ suspended in PBS containing 0.5% wt/vol Evans Blue Dye (6.5 × 10^7^ CFU per mouse) or PBS containing 0.5% wt/vol Evans Blue dye alone (control). Eighteen hours post-infection, blood, brain, GI tract, liver, lungs, and spleen were harvested aseptically and instilled in 70% ethanol. Slides were prepared and stained with hematoxylin and eosin by the Pathology EM and Histology Laboratory (UMB histology core facility) using standard protocols. Histopathological examination of the whole tissue was performed in a blinded fashion by an experienced pathologist. Histopathology was scored per Table 3 on a scale of 0–4, with 4 being the greatest degree of pathology for each parameter assessed.

**TABLE 3:** Scoring system to assess pathology of neonatal tissues 18 h post-infection^*a*^

Tissue	Parameter 1 scoring criteria	Parameter 2 scoring criteria	Parameter 3 scoring criteria	Maximum score
Brain	Increased apoptosis	Inflammatory cell infiltration	–^*b*^	8
Gastrointestinal tract	Necrosis to mid or entire villus	Inflammatory cell infiltration	Intestinal wall injury	12
Liver	Degradation and necrosis of hepatocytes	Infiltration of inflammatory cells, aggregation of inflammatory cells	–	8
Lung	Reduced alveolar vesicular structure	Airway inflammatory cell infiltration	–	8
Spleen	Increased number of apoptotic cells, granulocytes, macrophage aggregates, and inclusion bodies	Red blood cell depletion	Reduction in white pulp/architectural dysregulation	12

^*a*^ Pathology scored on a scale of 0–4. Each tissue was scored on the absence or severity of the listed parameters with brain, liver, and lungs having a maximum score of 8, and the gastrointestinal tract and spleen having a maximum score of 12. 0 = normal/absent, 1 = minimal, 2 = mild, 3 = moderate, and 4 = severe.

^*b*^ Dashes signify that parameter 3 was not applicable.

### Statistical analysis

All statistical analyses were performed with GraphPad Prism v.10.1.1 (La Jolla, CA). A *P* value equal to or below 0.05 was considered significant for each test. Survival analyses for Kaplan-Meier curves were accomplished by log-rank test. LD_50_ values were calculated by linear regression analysis. Statistical significance for recovered CFU after bacterial infection was assessed by two-tailed Mann-Whitney test. Histopathology significance was determined by Student’s *t*-test.

## Data Availability

All data underlying the results are within the article and its supporting information files.

## SUPPLEMENTAL MATERIAL

Fig. S1 spectrum.00697-25-s0001.png The 50% lethal dose of *K. pneumoniae* B5055 StrepR following peroral administration in neonatal CD-1 mice.

Fig. S2 spectrum.00697-25-s0002.png The 50% lethal dose of *K. pneumoniae* B5055 StrepR following intraperitoneal or subcutaneous administration in neonatal CD-1 mice.

Fig. S3 spectrum.00697-25-s0003.png The 50% lethal dose of *K. pneumoniae* B5055 StrepR following intraperitoneal or subcutaneous administration in neonatal C57BL/6 mice.

Fig. S4 spectrum.00697-25-s0004.png Survival comparison of C57BL/6 neonatal mice infected with clinical isolates of *K. pneumoniae*.

Fig. S5 spectrum.00697-25-s0005.png Assessment of infection methodology on bacterial burden in neonatal mice.

Fig. S6 spectrum.00697-25-s0006.png Pathology following peroral infection with *K. pneumoniae* B5055 StrepR in neonatal mice.

Fig. S7 spectrum.00697-25-s0007.png Image of peroral administration setup.

Supplemental material spectrum.00697-25-s0008.docx Supplemental figure legends.

ASM does not own the copyrights to Supplemental Material that may be linked to, or accessed through, an article. The authors have granted ASM a non-exclusive, world-wide license to publish the Supplemental Material files. Please contact the corresponding author directly for reuse.

## References

[1] Mukherjee S, Bhadury P, Mitra S, Naha S, Saha B, Dutta S, Basu S. 2023. Hypervirulent Klebsiella pneumoniae causing neonatal bloodstream infections: emergence of NDM-1-producing hypervirulent ST11-K2 and ST15-K54 strains possessing pLVPK-associated markers. Microbiol Spectr 11:e04121-22. doi:10.1128/spectrum.04121-22 36752639 PMC10101084

[2] Paczosa MK, Mecsas J. 2016. Klebsiella pneumoniae: going on the offense with a strong defense. Microbiol Mol Biol Rev 80:629–661. doi:10.1128/MMBR.00078-15 27307579 PMC4981674

[3] Choi M, Hegerle N, Nkeze J, Sen S, Jamindar S, Nasrin S, Sen S, Permala-Booth J, Sinclair J, Tapia MD, et al.. 2020. The diversity of lipopolysaccharide (O) and capsular polysaccharide (K) antigens of invasive Klebsiella pneumoniae in a multi-country collection. Front Microbiol 11:1249. doi:10.3389/fmicb.2020.01249 32595624 PMC7303279

[4] Khaertynov KS Khaertynov, Anokhin VA, Davidyuk YN, Nicolaeva IV, Khalioullina SV, Semyenova DR, Alatyrev EY, Skvortsova NN, Abrahamyan LG. 2017. Case of meningitis in a neonate caused by an extended-spectrum-beta-lactamase-producing strain of hypervirulent Klebsiella pneumoniae. Front Microbiol 8:1576. doi:10.3389/fmicb.2017.01576 28861076 PMC5559536

[5] Mezgebu T, Ossabo G, Zekiwos A, Mohammed H, Demisse Z. 2023. Neonatal sepsis and its associated factors among neonates admitted to the neonatal intensive care unit in Wachemo University Comprehensive Specialized Hospital, southern Ethiopia, 2022. Front Pediatr 11:1184205. doi:10.3389/fped.2023.1184205 37465417 PMC10350534

[6] Simonsen KA, Anderson-Berry AL, Delair SF, Davies HD. 2014. Early-onset neonatal sepsis. Clin Microbiol Rev 27:21–47. doi:10.1128/CMR.00031-13 24396135 PMC3910904

[7] Singh M, Alsaleem M, Gray CP. 2024. Neonatal sepsis. StatPearls Publishing, Treasure Island, FL. 30285373

[8] Wynn JL. 2016. Defining neonatal sepsis. Curr Opin Pediatr 28:135–140. doi:10.1097/MOP.0000000000000315 26766602 PMC4786443

[9] Okomo U, Akpalu ENK, Le Doare K, Roca A, Cousens S, Jarde A, Sharland M, Kampmann B, Lawn JE. 2019. Aetiology of invasive bacterial infection and antimicrobial resistance in neonates in sub-Saharan Africa: a systematic review and meta-analysis in line with the STROBE-NI reporting guidelines. Lancet Infect Dis 19:1219–1234. doi:10.1016/S1473-3099(19)30414-1 31522858

[10] Kumar CK, Sands K, Walsh TR, O’Brien S, Sharland M, Lewnard JA, Hu H, Srikantiah P, Laxminarayan R. 2023. Global, regional, and national estimates of the impact of a maternal Klebsiella pneumoniae vaccine: a Bayesian modeling analysis. PLoS Med 20:e1004239. doi:10.1371/journal.pmed.1004239 37216371 PMC10270628

[11] Russell NJ, Stöhr W, Plakkal N, Cook A, Berkley JA, Adhisivam B, Agarwal R, Ahmed NU, Balasegaram M, Ballot D, et al.. 2023. Patterns of antibiotic use, pathogens, and prediction of mortality in hospitalized neonates and young infants with sepsis: a global neonatal sepsis observational cohort study (NeoOBS). PLoS Med 20:e1004179. doi:10.1371/journal.pmed.1004179 37289666 PMC10249878

[12] Sands K, Carvalho MJ, Portal E, Thomson K, Dyer C, Akpulu C, Andrews R, Ferreira A, Gillespie D, Hender T, et al.. 2021. Characterization of antimicrobial-resistant Gram-negative bacteria that cause neonatal sepsis in seven low- and middle-income countries. Nat Microbiol 6:512–523. doi:10.1038/s41564-021-00870-7 33782558 PMC8007471

[13] Taylor AW, Blau DM, Bassat Q, Onyango D, Kotloff KL, Arifeen SE, Mandomando I, Chawana R, Baillie VL, Akelo V, et al.. 2020. Initial findings from a novel population-based child mortality surveillance approach: a descriptive study. Lancet Glob Health 8:e909–e919. doi:10.1016/S2214-109X(20)30205-9 32562647 PMC7303945

[14] Chaurasia S, Sivanandan S, Agarwal R, Ellis S, Sharland M, Sankar MJ. 2019. Neonatal sepsis in South Asia: huge burden and spiralling antimicrobial resistance. BMJ 364:k5314. doi:10.1136/bmj.k5314 30670451 PMC6340339

[15] Thomson KM, Dyer C, Liu F, Sands K, Portal E, Carvalho MJ, Barrell M, Boostrom I, Dunachie S, Farzana R, et al.. 2021. Effects of antibiotic resistance, drug target attainment, bacterial pathogenicity and virulence, and antibiotic access and affordability on outcomes in neonatal sepsis: an international microbiology and drug evaluation prospective substudy (BARNARDS). Lancet Infect Dis 21:1677–1688. doi:10.1016/S1473-3099(21)00050-5 34384533 PMC8612937

[16] WHO bacterial priority pathogens list, 2024: Bacterial pathogens of public health importance to guide research, development and strategies to prevent and control antimicrobial resistance. Available from: https://www.who.int/publications/i/item/9789240093461. Retrieved 17 Feb 2025. 10.1016/S1473-3099(25)00118-5 PMC12367593 40245910

[17] Assoni L, Girardello R, Converso TR, Darrieux M. 2021. Current stage in the development of Klebsiella pneumoniae vaccines. Infect Dis Ther 10:2157–2175. doi:10.1007/s40121-021-00533-4 34476772 PMC8412853

[18] Douradinha B. 2024. Exploring the journey: a comprehensive review of vaccine development against Klebsiella pneumoniae. Microbiol Res 287:127837. doi:10.1016/j.micres.2024.127837 39059097

[19] Miller JC, Cross AS, Tennant SM, Baliban SM. 2024. Klebsiella pneumoniae lipopolysaccharide as a vaccine target and the role of antibodies in protection from disease. Vaccines (Basel) 12:1177. doi:10.3390/vaccines12101177 39460343 PMC11512408

[20] Li J, Shen L, Qian K. 2023. Global, regional, and national incidence and mortality of neonatal sepsis and other neonatal infections, 1990-2019. Front Public Health 11:1139832. doi:10.3389/fpubh.2023.1139832 36998277 PMC10043440

[21] Cortese F, Scicchitano P, Gesualdo M, Filaninno A, De Giorgi E, Schettini F, Laforgia N, Ciccone MM. 2016. Early and late infections in newborns: where do we stand? A review. Pediatr Neonatol 57:265–273. doi:10.1016/j.pedneo.2015.09.007 26750406

[22] Cuenca AG, Wynn JL, Moldawer LL, Levy O. 2013. Role of innate immunity in neonatal infection. Am J Perinatol 30:105–112. doi:10.1055/s-0032-1333412 23297181 PMC3959733

[23] Kim F, Polin RA, Hooven TA. 2020. Neonatal sepsis. BMJ 371:m3672. doi:10.1136/bmj.m3672 33004379

[24] Assoni L, Couto AJM, Vieira B, Milani B, Lima AS, Converso TR, Darrieux M. 2024. Animal models of Klebsiella pneumoniae mucosal infections. Front Microbiol 15:1367422. doi:10.3389/fmicb.2024.1367422 38559342 PMC10978692

[25] Clements A, Jenney AW, Farn JL, Brown LE, Deliyannis G, Hartland EL, Pearse MJ, Maloney MB, Wesselingh SL, Wijburg OL, Strugnell RA. 2008. Targeting subcapsular antigens for prevention of Klebsiella pneumoniae infections. Vaccine (Auckland) 26:5649–5653. doi:10.1016/j.vaccine.2008.07.100 18725260

[26] Hegerle N, Choi M, Sinclair J, Amin MN, Ollivault-Shiflett M, Curtis B, Laufer RS, Shridhar S, Brammer J, Toapanta FR, Holder IA, Pasetti MF, Lees A, Tennant SM, Cross AS, Simon R. 2018. Development of a broad spectrum glycoconjugate vaccine to prevent wound and disseminated infections with Klebsiella pneumoniae and Pseudomonas aeruginosa. PLoS ONE 13:e0203143. doi:10.1371/journal.pone.0203143 30188914 PMC6126813

[27] Russo TA, MacDonald U, Hassan S, Camanzo E, LeBreton F, Corey B, McGann P. 2021. An assessment of siderophore production, mucoviscosity, and mouse infection models for defining the virulence spectrum of hypervirulent Klebsiella pneumoniae. mSphere 6:e00045-21. doi:10.1128/mSphere.00045-21 33762316 PMC8546679

[28] Bernardini R, Aufieri R, Detcheva A, Recchia S, Cicconi R, Amicosante M, Montesano C, Rossi P, Tchidjou HK, Petrunov B, Orefici G, Mattei M. 2017. Neonatal protection and preterm birth reduction following maternal group B streptococcus vaccination in a mouse model. J Matern Fetal Neonatal Med 30:2844–2850. doi:10.1080/14767058.2016.1265932 27973991

[29] Buurman ET, Timofeyeva Y, Gu J, Kim J-H, Kodali S, Liu Y, Mininni T, Moghazeh S, Pavliakova D, Singer C, Singh S, Handke LD, Lotvin J, Prasad AK, Scully IL, Donald RGK, Jansen KU, Anderson AS. 2019. A novel hexavalent capsular polysaccharide conjugate vaccine (GBS6) for the prevention of neonatal group B streptococcal infections by maternal immunization. J Infect Dis 220:105–115. doi:10.1093/infdis/jiz062 30778554 PMC6548902

[30] Dalgakiran F, Witcomb LA, McCarthy AJ, Birchenough GMH, Taylor PW. 2014. Non-invasive model of neuropathogenic Escherichia coli infection in the neonatal rat. J Vis Exp 52018:e52018. doi:10.3791/52018 PMC4353393 25408299

[31] Sereme Y, Schrimp C, Faury H, Agapoff M, Lefebvre-Wloszczowski E, Chang Marchand Y, Ageron-Ardila E, Panafieu E, Blec F, Coureuil M, Frapy E, Tsatsaris V, Bonacorsi S, Skurnik D. 2024. A live attenuated vaccine to prevent severe neonatal Escherichia coli K1 infections. Nat Commun 15:3021. doi:10.1038/s41467-024-46775-x 38589401 PMC11001983

[32] Cross AS, Opal SM, Warren HS, Palardy JE, Glaser K, Parejo NA, Bhattacharjee AK. 2001. Active immunization with a detoxified Escherichia coli J5 lipopolysaccharide group B meningococcal outer membrane protein complex vaccine protects animals from experimental sepsis. J Infect Dis 183:1079–1086. doi:10.1086/319297 11237833

[33] Liu L, Maharjan S, Sun J-L, Li Y-C, Cheng H-J. 2021. Prevalence and clinical characteristics of septicemia in children with Mycoplasma pneumoniae pneumonia. J Int Med Res 49:3000605211021733. doi:10.1177/03000605211021733 34167353 PMC8236790

[34] Mahmoud HAH, Parekh R, Dhandibhotla S, Sai T, Pradhan A, Alugula S, Cevallos-Cueva M, Hayes BK, Athanti S, Abdin Z. 2023. Insight into neonatal sepsis: an overview. Cureus 15:e45530. 37868444 10.7759/cureus.45530 PMC10585949

[35] Saini K, Bolia R, Bhat NK. 2022. Incidence, predictors and outcome of sepsis-associated liver injury in children: a prospective observational study. Eur J Pediatr 181:1699–1707. doi:10.1007/s00431-022-04374-2 35020050 PMC8753337

[36] You T, Zhou Y-R, Liu X-C, Li L-Q. 2022. Risk factors and clinical characteristics of neonatal acute respiratory distress syndrome caused by early onset sepsis. Front Pediatr 10:847827. doi:10.3389/fped.2022.847827 35419326 PMC8995893

[37] Esposito AL, Pennington JE. 1983. Effects of aging on antibacterial mechanisms in experimental pneumonia. Am Rev Respir Dis 128:662–667. doi:10.1164/arrd.1983.128.4.662 6354025

[38] Ashurst JV, Dawson A. 2024. Klebsiella pneumonia. StatPearls Publishing, Treasure Island, FL. 30085546

[39] Crellen T, Turner P, Pol S, Baker S, Nguyen Thi Nguyen T, Stoesser N, Day NP, Turner C, Cooper BS. 2019. Transmission dynamics and control of multidrug-resistant Klebsiella pneumoniae in neonates in a developing country. Elife 8:e50468. doi:10.7554/eLife.50468 31793878 PMC6977969

[40] Young TM, Bray AS, Nagpal RK, Caudell DL, Yadav H, Zafar MA. 2020. Animal model to study Klebsiella pneumoniae gastrointestinal colonization and host-to-host transmission. Infect Immun 88:e00071-20. doi:10.1128/IAI.00071-20 32839189 PMC7573435

[41] Bonfanti P, Bellù R, Principe L, Caramma I, Condò M, Giani T, Rossolini GM, Luzzaro F. 2017. Mother-to-child transmission of KPC carbapenemase-producing Klebsiella pneumoniae at birth. Pediatr Infect Dis J 36:228–229. doi:10.1097/INF.0000000000001403 27846056

[42] Danino D, Melamed R, Sterer B, Porat N, Hazan G, Gushanski A, Shany E, Greenberg D, Borer A. 2018. Mother-to-child transmission of extended-spectrum-beta-lactamase-producing Enterobacteriaceae. J Hosp Infect 100:40–46. doi:10.1016/j.jhin.2017.12.024 29330015

[43] Kumar M, Saadaoui M, Al Khodor S. 2022. Infections and pregnancy: effects on maternal and child health. Front Cell Infect Microbiol 12:873253. doi:10.3389/fcimb.2022.873253 35755838 PMC9217740

[44] Schwartz DJ, Shalon N, Wardenburg K, DeVeaux A, Wallace MA, Hall-Moore C, Ndao IM, Sullivan JE, Radmacher P, Escobedo M, Burnham C-A, Warner BB, Tarr PI, Dantas G. 2023. Gut pathogen colonization precedes bloodstream infection in the neonatal intensive care unit. Sci Transl Med 15:eadg5562. doi:10.1126/scitranslmed.adg5562 37134153 PMC10259202

[45] Sun Y, Patel A, SantaLucia J, Roberts E, Zhao L, Kaye K, Rao K, Bachman MA. 2021. Measurement of Klebsiella intestinal colonization density to assess infection risk. mSphere 6:e00500-21. doi:10.1128/mSphere.00500-21 34160234 PMC8265666

[46] Verani JR, Blau DM, Gurley ES, Akelo V, Assefa N, Baillie V, Bassat Q, Berhane M, Bunn J, Cossa ACA, et al.. 2024. Child deaths caused by Klebsiella pneumoniae in sub-Saharan Africa and south Asia: a secondary analysis of Child Health and Mortality Prevention Surveillance (CHAMPS) data. Lancet Microbe 5:e131–e141. doi:10.1016/S2666-5247(23)00290-2 38218193 PMC10849973

[47] Mao Q, Wang Y, Gao R, Shao J, Yao X, Lang S, Wang C, Mao P, Liang Z, Wang J. 2012. A neonatal mouse model of coxsackievirus A16 for vaccine evaluation. J Virol 86:11967–11976. doi:10.1128/JVI.00902-12 22951825 PMC3486452

[48] De Freitas Caires N, Gaudet A, Portier L, Tsicopoulos A, Mathieu D, Lassalle P. 2018. Endocan, sepsis, pneumonia, and acute respiratory distress syndrome. Crit Care 22:280. doi:10.1186/s13054-018-2222-7 30367649 PMC6204032

[49] Wohlrab P, Soto-Gonzales L, Benesch T, Winter MP, Lang IM, Markstaller K, Tretter V, Klein KU. 2018. Intermittent hypoxia activates duration-dependent protective and injurious mechanisms in mouse lung endothelial cells. Front Physiol 9:1754. doi:10.3389/fphys.2018.01754 30574096 PMC6291480

[50] You T, Zhang H, Guo L, Ling K-R, Hu X-Y, Li L-Q. 2020. Differences in clinical characteristics of early- and late-onset neonatal sepsis caused by Klebsiella pneumoniae. Int J Immunopathol Pharmacol 34:2058738420950586. doi:10.1177/2058738420950586 32816593 PMC7444108

[51] Siu LK, Yeh K-M, Lin J-C, Fung C-P, Chang F-Y. 2012. Klebsiella pneumoniae liver abscess: a new invasive syndrome. Lancet Infect Dis 12:881–887. doi:10.1016/S1473-3099(12)70205-0 23099082

[52] Russo TA, Marr CM. 2019. Hypervirulent Klebsiella pneumoniae. Clin Microbiol Rev 32:e00001-19. doi:10.1128/CMR.00001-19 31092506 PMC6589860

[53] Holt KE, Wertheim H, Zadoks RN, Baker S, Whitehouse CA, Dance D, Jenney A, Connor TR, Hsu LY, Severin J, et al.. 2015. Genomic analysis of diversity, population structure, virulence, and antimicrobial resistance in Klebsiella pneumoniae, an urgent threat to public health. Proc Natl Acad Sci USA 112:E3574–81. doi:10.1073/pnas.1501049112 26100894 PMC4500264

[54] Trautmann M, Ruhnke M, Rukavina T, Held TK, Cross AS, Marre R, Whitfield C. 1997. O-antigen seroepidemiology of Klebsiella clinical isolates and implications for immunoprophylaxis of Klebsiella infections. Clin Diagn Lab Immunol 4:550–555. doi:10.1128/cdli.4.5.550-555.1997 9302204 PMC170594

