# Supplementary figures and images for: Development of a mouse model for Klebsiella pneumoniae-associated neonatal sepsis
Source: Microbiol Spectr. 2025 Aug 1;13(9):e00697-25. doi: 10.1128/spectrum.00697-25 (PMC12403570; doi:10.1128/spectrum.00697-25)

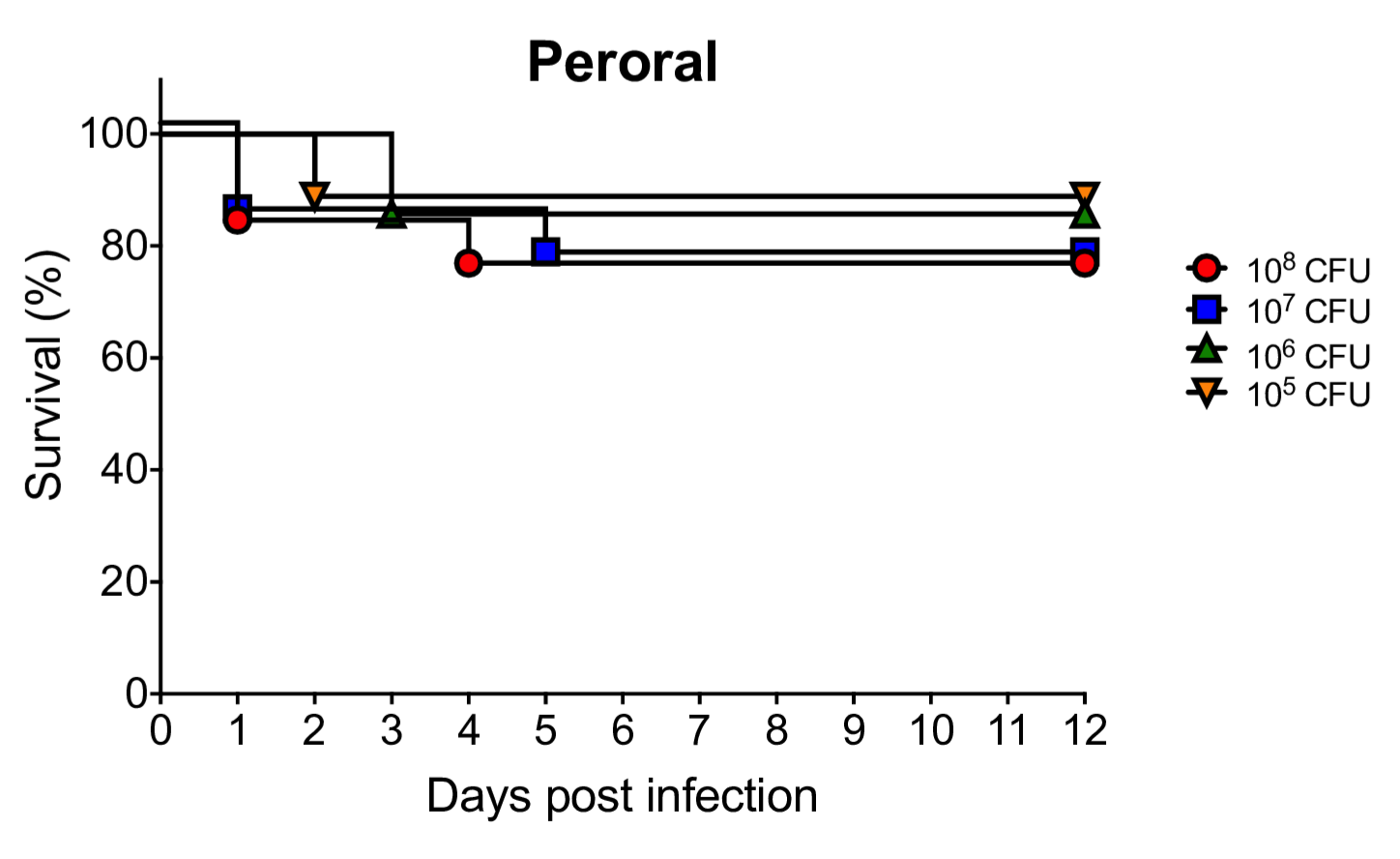

Supplement: Fig. S1 — The 50% lethal dose of K. pneumoniae B5055 StrepR following peroral administration in neonatal CD-1 mice. [file spectrum.00697-25-s0001.png]

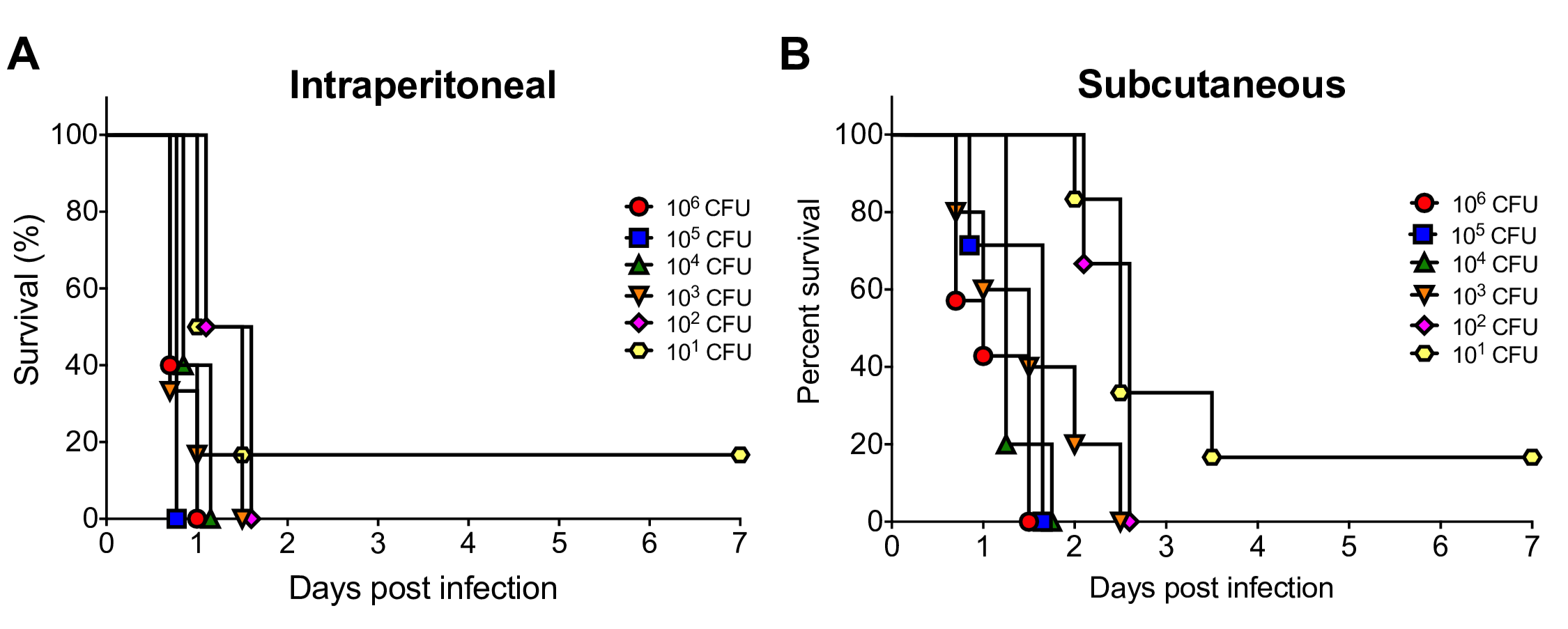

Supplement: Fig. S2 — The 50% lethal dose of K. pneumoniae B5055 StrepR following intraperitoneal or subcutaneous administration in neonatal CD-1 mice. [file spectrum.00697-25-s0002.png]

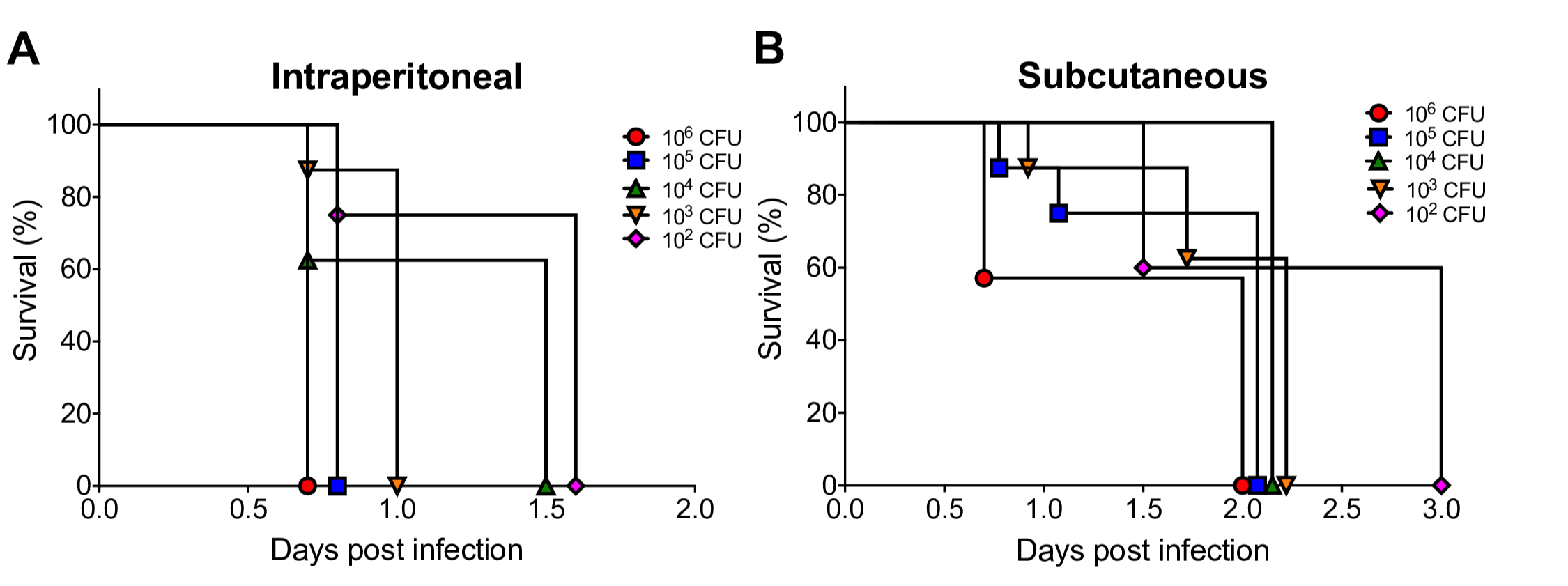

Supplement: Fig. S3 — The 50% lethal dose of K. pneumoniae B5055 StrepR following intraperitoneal or subcutaneous administration in neonatal C57BL/6 mice. [file spectrum.00697-25-s0003.png]

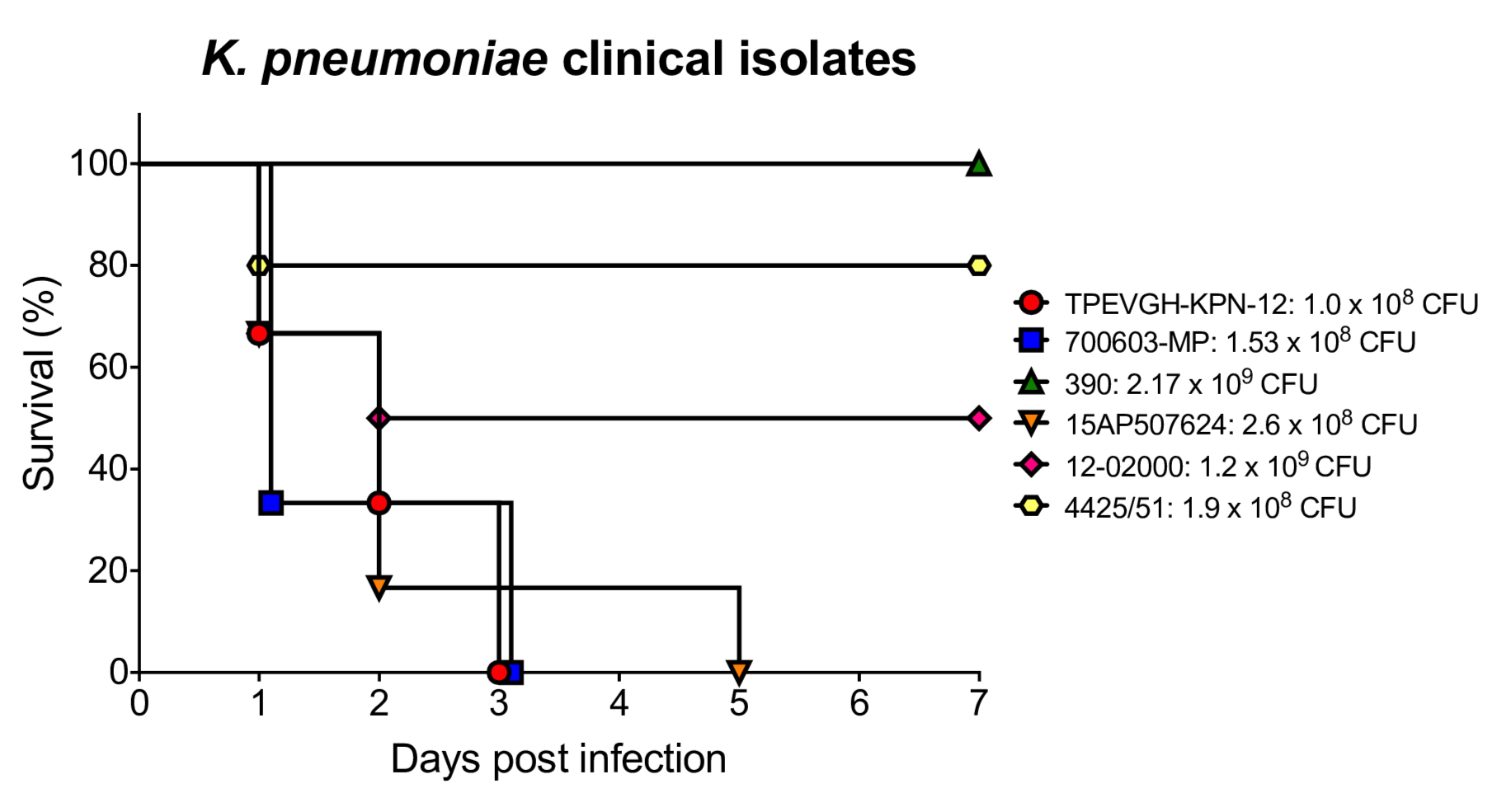

Supplement: Fig. S4 — Survival comparison of C57BL/6 neonatal mice infected with clinical isolates of K. pneumoniae. [file spectrum.00697-25-s0004.png]

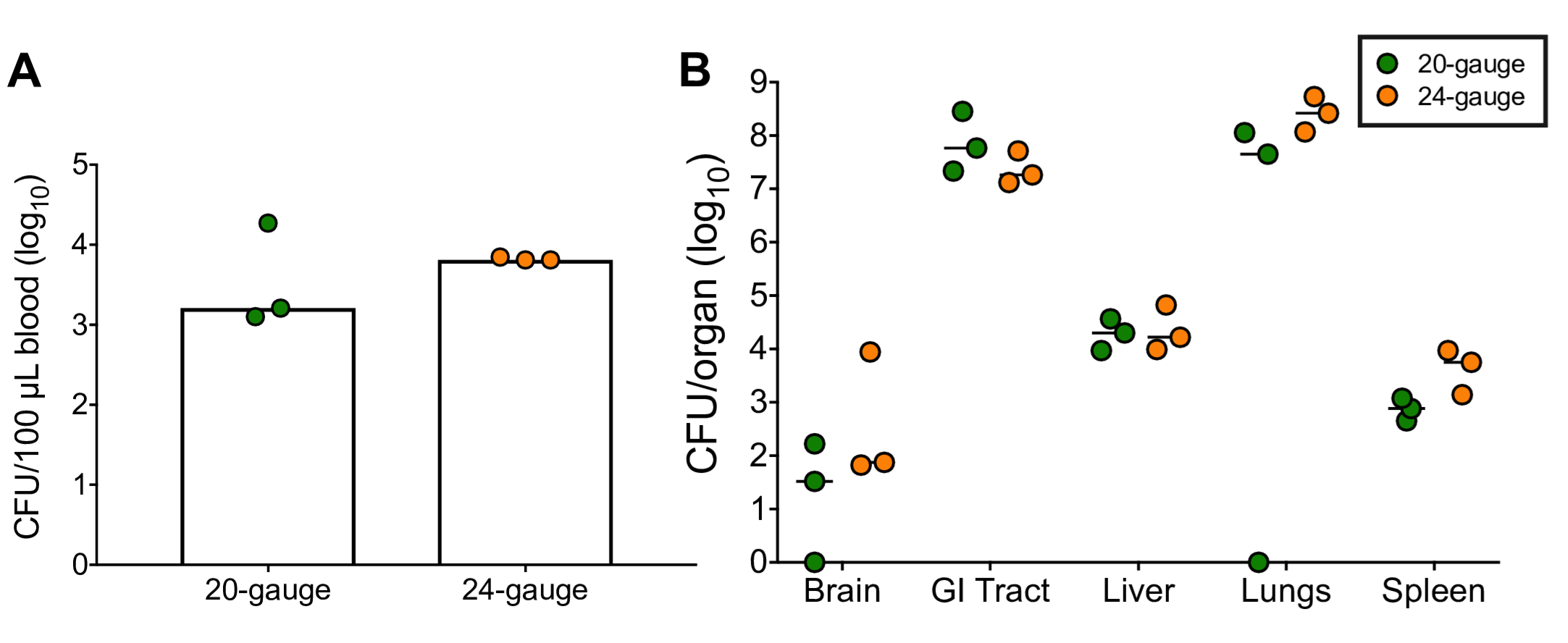

Supplement: Fig. S5 — Assessment of infection methodology on bacterial burden in neonatal mice. [file spectrum.00697-25-s0005.png]

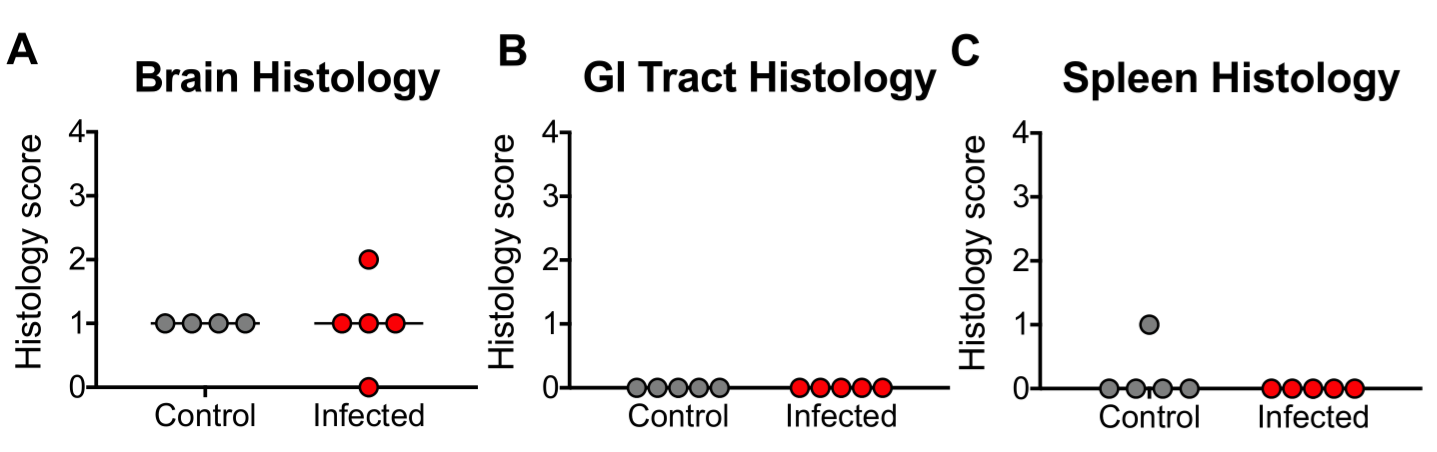

Supplement: Fig. S6 — Pathology following peroral infection with K. pneumoniae B5055 StrepR in neonatal mice. [file spectrum.00697-25-s0006.png]

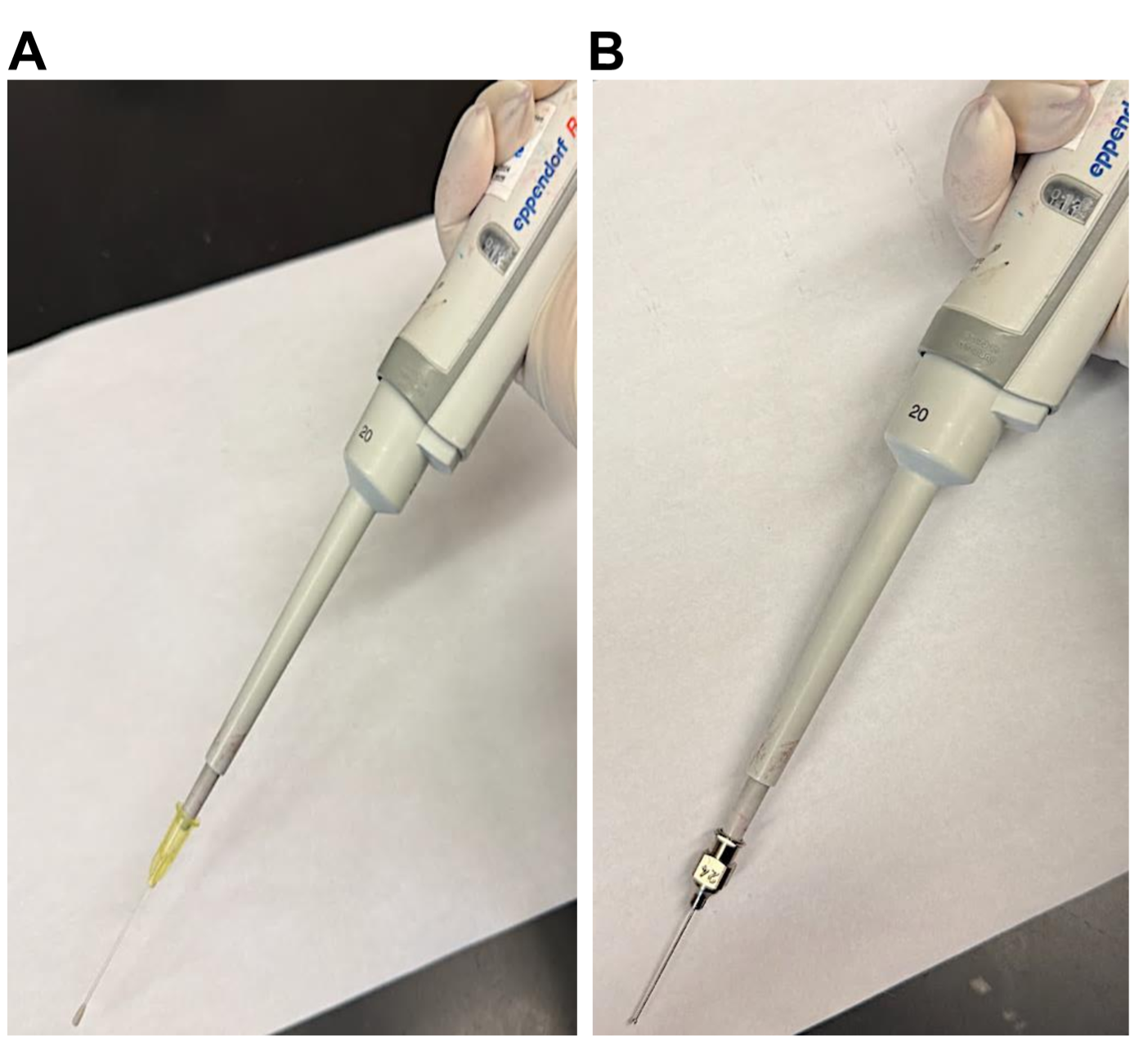

Supplement: Fig. S7 — Image of peroral administration setup. [file spectrum.00697-25-s0007.png]
