# Supplementary material for: Development of a mouse model for Klebsiella pneumoniae-associated neonatal sepsis
Source: Microbiol Spectr. 2025 Aug 1;13(9):e00697-25. doi: 10.1128/spectrum.00697-25 (PMC12403570; doi:10.1128/spectrum.00697-25)
Supplement: Supplemental material — Supplemental figure legends. [file spectrum.00697-25-s0008.docx]

**Figure S1. The 50% lethal dose of *K. pneumoniae* B5055 Strep^R^ following peroral administration in neonatal CD-1 mice.** Whole litters of two- and three-day-old CD-1 mice (n = 6-15 per litter) were infected perorally with the indicated inocula of *K. pneumoniae* B5055 Strep^R^ and monitored daily for survival. The Kaplan-Meier survival curves shown are one representative experiment performed at least two times.

**Figure S2. The 50% lethal dose of *K. pneumoniae* B5055 Strep^R^ following intraperitoneal or subcutaneous administration in neonatal CD-1 mice.** Whole litters of two-day-old CD-1 mice (n = 5-7 per litter) were split and either infected (**A**) intraperitoneally or (**B**) subcutaneously with the indicated inocula of *K. pneumoniae* B5055 Strep^R^ and monitored daily for survival. The Kaplan-Meier survival curves shown are a single experiment for each administration route.

**Figure S3. The 50% lethal dose of *K. pneumoniae* B5055 Strep^R^ following intraperitoneal or subcutaneous administration in neonatal C57BL/6 mice.** Whole litters of two-day-old C57BL/6 mice (n = 4-8 per litter) were infected (**A**) intraperitoneally or (**B**) subcutaneously with the indicated inocula of *K. pneumoniae* B5055 Strep^R^ and monitored daily for survival. The Kaplan-Meier survival curves shown are a single experiment for each administration route.

**Figure S4. Survival comparison of C57BL/6 neonatal mice infected with clinical isolates of *K. pneumoniae*.** Two-day-old C57BL/6 (n = 3-6 per group) were infected perorally with the indicated inocula of *K. pneumoniae* TPEVGH-KPN-12, 700603-MP, 390, 15AP507624, 12-02000, or 4425/51 and monitored daily for survival. The Kaplan-Meier survival curves shown are a single experiment for each clinical isolate.

**Figure S5. Assessment of infection methodology on bacterial burden in neonatal mice.** Two-day-old C57BL/6 mice (n = 3 per group) were infected perorally with 1.5 x 10^7^ CFU of *K. pneumoniae* B5055 Strep^R^ using either a PFTE 20-gauge or a stainless steel 24-gauge feeding needles. Bacterial loads were determined in the (**A**) blood and (**B**) tissues (brain, GI tract, liver, lungs, and spleen) at 2 h.p.i. Each point represents an individual mouse. Median burden is represented by a bar or line.

**Figure S6. Pathology following peroral infection with *K. pneumoniae* B5055 Strep^R^ in neonatal mice.** (**A**) Brain, (**B**) GI tract, and (**C**) spleen pathology were scored in *K. pneumoniae* B5055 Strep^R^-infected and control two-day-old C57BL/6 mice (n = 5 per group) at 18 h.p.i. Degree of pathology was based on Table 2, with a maximum score of 12. Data points represent an individual mouse from a single experiment.

**Figure S7. Image of peroral administration setup.** Neonatal mice were infected perorally with a 10 μL volume of *K. pneumoniae* B5055 Strep^R^ suspended in PBS containing 0.5% w/v Evans blue dye. The inoculum was administered using a 20 μL fixed volume pipette attached to either (**A**) a flexible, PTFE 20-gauge x 1.5” feeding needle with 2 mm ball or (**B**) a straight, stainless steel 24-gauge x 1” feeding needle with 1.25 mm ball.
